# Prevalence of autoimmune pancreatitis in pancreatic resection for suspected malignancy: a systematic review and meta-analysis

**DOI:** 10.1186/s12876-024-03367-9

**Published:** 2024-08-21

**Authors:** Zain A. Karamya, Attila Kovács, Dóra Illés, Bálint Czakó, Alíz Fazekas, Nelli Farkas, Péter Hegyi, László Czakó

**Affiliations:** 1 Division of Gastroenterology, Department of Medicine, University of Szeged, Pf.: 427, Szeged, H-6701 Hungary; 2 Department of Gastroenterology and Internal Medicine, Markusovszky Teaching Hospital, Szombathely, Hungary; 3 Institute for Translational Medicine, Medical School, University of Pécs, Pécs, Hungary; 4 Institute of Bioanalysis, Medical School, University of Pécs, Pécs, Hungary; 5 Division of Pancreatic Diseases, Heart and Vascular Centre, Semmelweis University, Budapest, Hungary

**Keywords:** Autoimmune pancreatitis, Pancreaticoduodenectomy, Pancreatic cancer, Pancreatic resection, IgG4, Whipple’s procedure

## Abstract

**Background/Objectives:**

Autoimmune pancreatitis (AIP) is a diagnosis-challenging disease that often mimics pancreatic malignancy. Pancreatic resection is considered to be a curative treatment for pancreatic ductal adenocarcinoma (PDAC). This meta-analysis aims to study the incidence of AIP in patients who have undergone pancreatic resection for clinical manifestation of cancer.

**Methods:**

A comprehensive search was conducted in three databases, PubMed, Embase and the Cochrane Library, using the terms ‘autoimmune pancreatitis’ and ‘pancreatic resection’ and supplemented by manual checks of reference lists in all retrieved articles.

**Results:**

Ten articles were included in the final analysis. 8917 pancreatic resections were performed because of a clinical suspicion of pancreatic cancer. AIP accounted for 140 cases (1.6%). Type 1 AIP comprised the majority of cases, representing 94% (132 cases), while type 2 AIP made up the remaining 6% (eight cases) after further classification. AIP accounted for almost 26% of all cases of benign diseases involving unnecessary surgery and was overrepresented in males in 70% of cases compared to 30% in females. The mean age for AIP patients was 59 years. Serum CA 19 − 9 levels were elevated in 23 out of 47 (49%) AIP patients, where higher levels were detected more frequently in patients with type 1 AIP (51%, 22 out of 43) than in those with type 2 AIP (25%, 1 out of 4). The sensitivity of IgG4 levels in type 1 AIP was low (43%, 21/49 patients).

**Conclusion:**

Even with modern diagnostic methods, distinguishing between AIP and PDAC can still be challenging, thus potentially resulting in unnecessary surgical procedures in some cases. Serum CA 19 − 9 levels are not useful in distinguishing between AIP and PDAC. Work must thus be done to improve diagnostic methods and avoid unnecessary complicated surgery.

## Background

Autoimmune pancreatitis (AIP) is a rare disease that was first reported by Sarles et al. in 1961 [1]. AIP can be histologically divided into two types: type 1 AIP, or lymphoplasmacytic sclerosing pancreatitis (LPSP), and type 2 AIP, or idiopathic duct-centric pancreatitis (IDCP). AIP can present with abdominal pain, jaundice, weight loss and fatigue. LPSP can be distinguished by dense infiltration of plasma cells and lymphocytes and abundant (> 10 cells per high-power field) immunoglobulin (Ig) G4 positive plasma cells. In contrast, IDCP demonstrates notable neutrophilic inflammation, often leading to the destruction and obliteration of the duct lumen. Moreover, unlike LPSP, IDCP is a pancreas-specific disorder not associated with elevated serum IgG4 or involving other organs [2].

The diagnostic work-up for AIP can be challenging [3]. Perhaps the greatest challenge for clinicians in diagnosing AIP is that it can frequently mimic pancreatic adenocarcinoma (PDAC), subsequently leading to unnecessary surgery. Despite all the improvement in the diagnostic work-up, clinicians sometimes find it difficult to detect the precise cause behind the pancreatic lesion; they will thus face the challenge of whether to choose surgery for a non-neoplastic disease or conservative treatment for a potentially lethal cancer. Furthermore, PDAC is more frequent in AIP patients, making the differential diagnosis even more challenging [4].

Pancreatic resection (distal or total pancreatectomy and pancreaticoduodenectomy, also known as Whipple’s procedure) is considered a potentially curative treatment for PDAC [5]. Despite the advancement of medical care, mortality and morbidity percentages of pancreatectomy are still high, with some studies reporting morbidity of 46% [6]. Therefore, it is advisable to perform pancreatic resection when there is a clear indication.

This meta-analysis aims to evaluate the frequency of AIP in pancreatic resections performed for a clinical suspicion of pancreatic malignancy.

Methods.

## Search strategy

This study was conducted according to the principles in Preferred Reporting Items for Systematic Reviews and Meta Analyses. The study was recorded in the PROSPERO registry with the registration number CRD42023491749. A systematic search was made in three databases, PubMed, Embase and the Cochrane Library, with the following terms: pancreaticoduodenectomy [all fields] or Whipple [all fields] and (autoimmune pancreatitis [all fields]) and (humans [MeSHterms] and English [lang]). The Mendeley Reference Manager ^®^ (Elsevier, the Netherlands) was used to remove duplicates.

All full-text English-language articles with human data that reported the prevalence of AIP in pancreatic surgical resections performed for a clinical suspicion of pancreatic malignancy were included.

Exclusion criteria were the following: systematic reviews, review articles, single case reports, letters of correspondence and editorials; data repeated from previously published articles; and studies reporting on non-consecutive patients.

### Study selection

The studies were selected separately by two investigators (ZAK and LC). Clinical studies were eligible if they reported the occurrence of AIP in the histological analysis of a resected specimen from patients that had undergone pancreatic resection for suspicion of pancreatic malignancy. The reference lists in the articles obtained were also checked, but no additional eligible articles were found.

### Data synthesis and analysis

Proportion with 95% confidence interval (CI) was used for the effect size measure. To calculate the study proportions and pooled proportion, the total number of patients and those with the event of interest was extracted from each study.

Random intercept logistic regression model method was used to pool proportions (as recommended by Schwarzer et al. [7] and Stijnen et al. [8]. Hartung-Knapp adjustment [9, 10] was used for CIs calculation. The prediction intervals (i.e. the expected range of effects of future studies) was reported as well, it was calculated based on t-distribution.

Between-study heterogeneity, I2 statistics was determined as described by the Higgins & Thompson’s [11]. All statistical analyses were made with R (R Core Team 2023, v4.3.0) using the meta (Schwarzer 2023, v6.2.1) package for basic meta-analysis calculations and plots, and dmetar (Cuijpers, Furukawa, and Ebert 2022, v0.0.9000) package for additional influential analysis calculations and plots.

### Quality of studies and risk of Bias

The Newcastle-Ottawa scale, a star-based system, was employed to evaluate the quality of nonrandomized cohort studies [12]. This assessment focused on three key aspects: study selection, comparability of groups, and outcome data. Items deemed high-quality, with a low risk of bias, were awarded one star, while low-quality items, carrying a high or unknown risk of bias, received no stars (See Tables 1 and 2). Publication bias was assessed with funnel plot and tested with Egger’s test (See Figs. 4 and 5).

**Table 1:** Modified Newcastle–Ottawa Scale Criteria

	Adapted Newcastle-Ottawa Scale Items	High-quality Items Carrying a Low Risk of Bias (Green)	Low-quality Items Carrying a High (Red) or an Unknown (Yellow) Risk of Bias
Selection	**Item 1**: Representativeness of the initial study population – Patients with suspected malignancy and a final diagnosis of a benign disease	All patients with clinical suspicion of pancreatic cancer and their final histological diagnosis are benign disease were included	Low: any selection criteria were applied to the study population. Unknown: no data on selection process.
	**Item 2**: Representativeness of the initial study population – Patients with suspected malignancy and a final diagnosis of pancreatic cancer	All patients with a final histological diagnosis of malignancy were included.	Low: any selection criteria were applied to the study population. Unknown: no data on selection process.
	**Item 3**: Demonstration that outcome of interest was not present at start of study	Patients were presented with clinical symptoms of pancreatic cancer; their imaging were also indicative of cancer and no signs of autoimmune disease	Low: patients with pre-existing autoimmune disease or family history of AIP. Unknown: no statement.
Comparability	**Item 4**: study control for sex	No significant difference was detected between male/female patients regarding AIP	Low: significant difference was detected between male/female patients regarding AIP Unknown: no data was reported regarding sex.
	**Item 5**: Study control for age	No significant difference was detected between AIP and PDAC patients regarding age	Low: significant difference was detected between AIP and PDAC patients regarding age. Unknown: no data was reported regarding age.
Outcome	**Item 6**: Adequacy of histology report	Complete histology study reporting the final diagnosis after surgery	Low: incomplete histology study after surgery. Unknown: no reports on final diagnosis after surgery.

**Table 2:** Stars-rating based on the Modified Newcastle–Ottawa Scale

Aritcle	Item 1	Item 2	Item 3	Item 4	Item 5	Item 6	Total
Wojcicki, 2015	*	*	*	**-**	**-**	*	4
van Heerde, 2012	*	*	*	*	*	*	6
Räty, 2015	*	**-**	*	**-**	**-**	*	3
Chuong T. Tran, 2012	*	*	*	**-**	**-**	*	4
Jiang, 2017	*	*	*	**-**	**-**	*	4
Abraham, 2003	*	*	**-**	**-**	**-**	*	3
Vitali, 2014	*	*	*	**-**	**-**	*	4
de Castro, 2009	*	*	**-**	**-**	**-**	*	3
Yarandi,2014	*	**-**	*	*	**-**	*	4
Javed, 2021	*	**-**	**-**	**-**	**-**	*	2

## Results

**Fig. 1:**
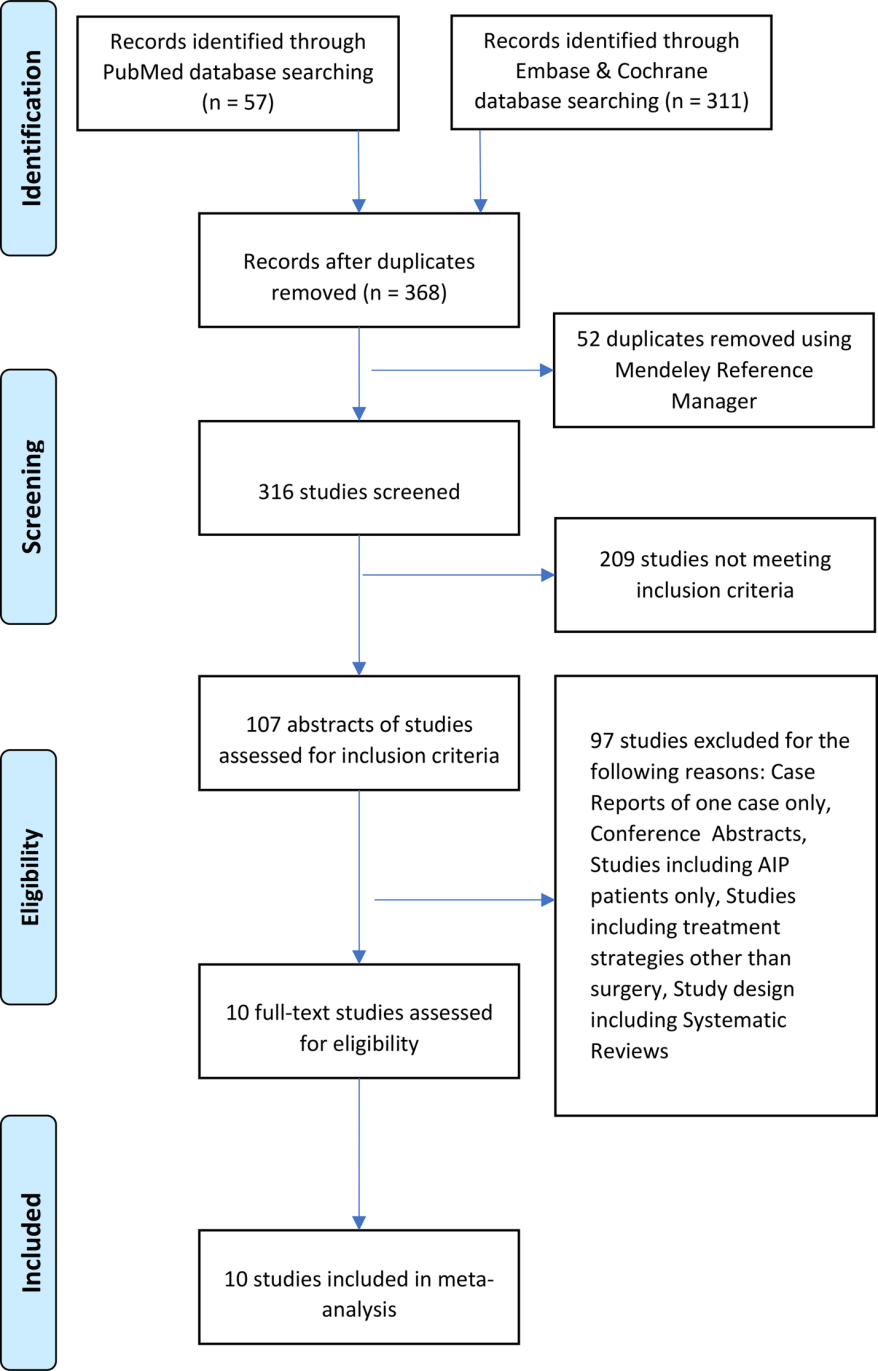
Study search and selection diagram

Database searches produced a total of 368 articles between 2001 and 2022 (Fig. 1). Out of 107 studies, only ten full articles were reviewed in full length and were later included in the final analysis [6, 13–21]. 97 studies were discarded because they were irrelevant to our research aim.

According to the ten studies, 8917 pancreatectomies were conducted between 1987 and 2016 due to clinical suspicion of pancreatic cancer. All included articles provided the total number of patients who had undergone pancreatectomies and then the number of cases in which the histopathological examination revealed a benign disease (Table 3).

**Table 3:** Characteristics and details of the ten included studies

First author	Study details (type, location, centre, period)	Sample size	Number of benign cases	AIP patients		Male/female (AIP patients)	Age (years)
				Type 1	Type 2		
Wojcicki, 2015 [20]	Retrospective, UK, single centre, Jan. 2004 to Dec. 2010	469	34	8	1	NR	NR
van Heerde, 2012 [18]	Retrospective, Netherlands, single centre, Jan. 2000 to Jan. 2009	274	36	3	4	6/1	Mean 53
Räty, 2015 [16]	Retrospective, Finland, single centre, 1987 to 2009	33	10	10	0	NR	NR
Chuong T. Tran, 2012 [17]	Retrospective, Honolulu, single centre, 2000 to 2010	65	3	3	0	NR	NR
Jiang, 2017 [15]	Retrospective, Canada, single centre, Feb. 2014 to Aug. 2016	40	3	3	0	3	Mean 63.6
Abraham, 2003 [13]	Retrospective, USA, single centre, Jan. 1999 to June 2001	442	47	11	0	8/3	Mean 57.1
Vitali, 2014 [19]	Retrospective, Germany, multicentre, Jan. 2005 to Sept. 2011	373	33	8	3	NR	NR
de Castro, 2009 [6]	Retrospective, Netherlands, single centre, Jan. 1992 to Dec. 2005	639	63	24	0	NR	NR
Yarandi, 2014 [21]	Retrospective, USA, single centre, Jan. 1998 to Dec. 2011	878	95	6	0	NR	NR
Javed, 2021 [14]	Retrospective, USA, single centre, 2001 to 2016	5709	NR	56	0	37/19	Mean 61.9

NR: not reported

**Fig. 2:**
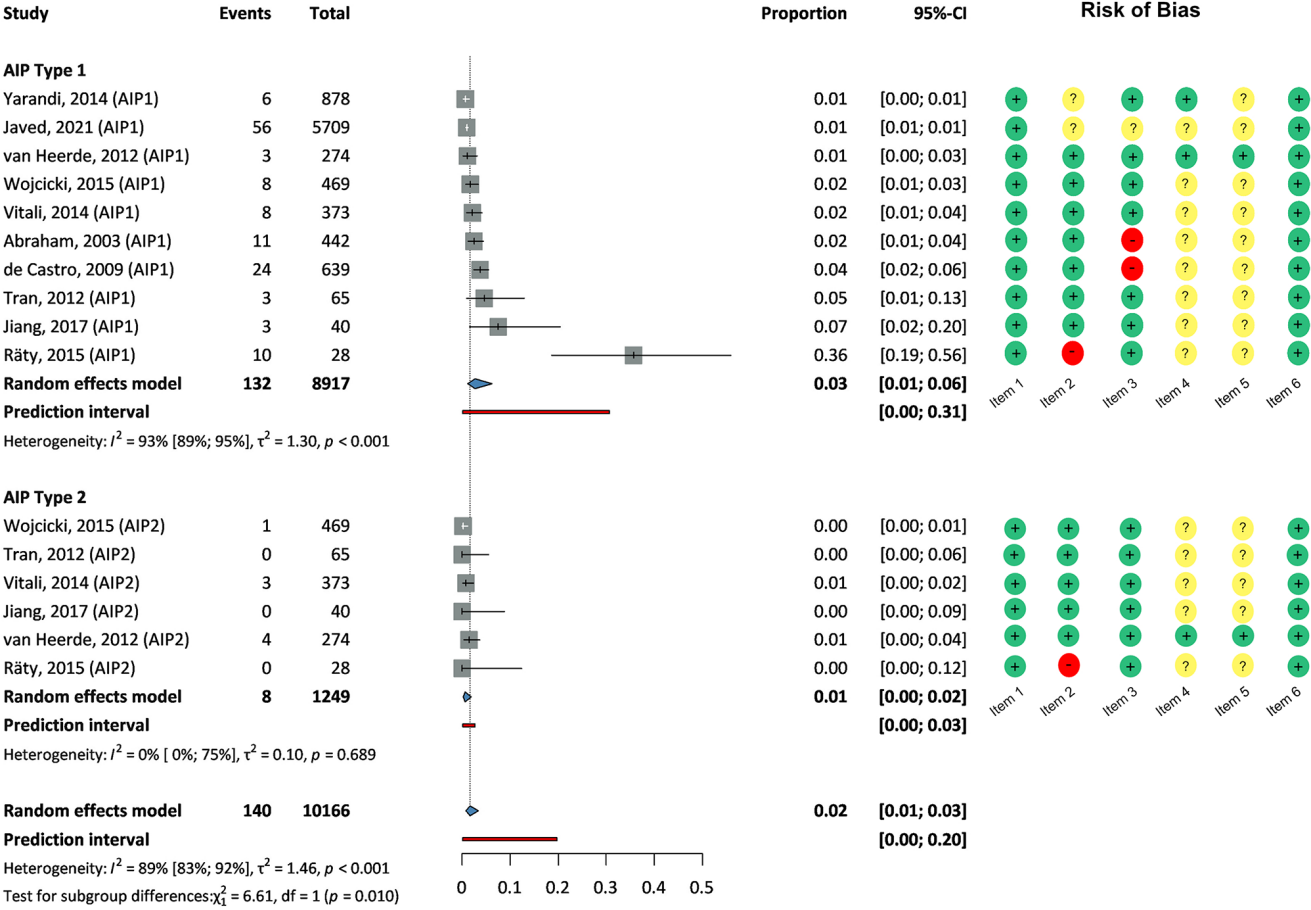
Forrest plots depicting the prevalence of type 1 and type 2 autoimmune pancreatitis in patients undergoing resection due to suspected pancreatic cancer. Size of squares for the proportion reflects the weight of the trial in the pooled analysis. The diamonds show the pooled prevalence of the types. Horizontal bars represent 95% CI. Red lines show the prediction interval

140 patients out of 8917 pancreatectomies (1.6%) were diagnosed with either type 1 or type 2 AIP. The overall proportion of these cases was 0.02 with a high level of confidence (95% CI: 0.01–0.03) using a statistical model that considers variability between studies. For type 1 AIP, the proportion was 0.03 (95% CI: 0.01–0.06) by subgroup analyses, indicating substantial diversity between studies (heterogeneity: 93%, CI: 89-96%). The prediction interval, representing where the true proportion of a randomly selected population could fall, ranged from 0 to 0.31. For type 2 AIP the proportion was 0.01 (95% CI: 0-0.02), with low heterogeneity (0%, CI: 0-75%). The prediction interval was 0.03 to 0.17. A statistical test revealed a significant difference between these subgroups (*p* = 0.01) (See Fig. 2).

From the 324 benign cases, 84 were (26%) diagnosed with type 1 or type 2 AIP. In this subgroup analysis, the proportion was 0.2 (95% CI: 0.07–0.48), with moderate diversity between studies (heterogeneity: 59%, CI:27-77%). The prediction interval ranged from 0 to 0.97.

**Fig. 3:**
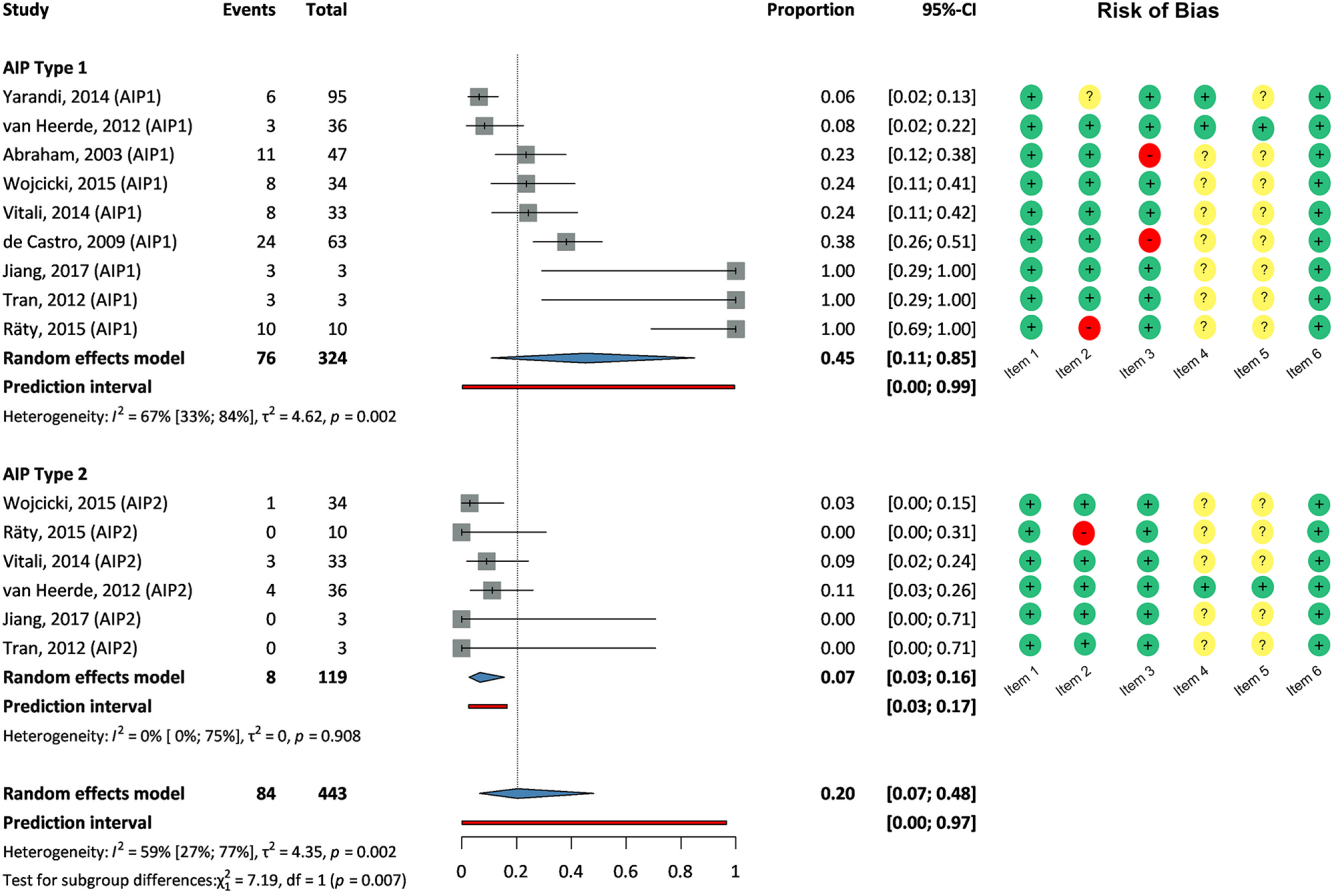
Forrest plots depicting the prevalence of type 1 and type 2 autoimmune pancreatitis in patients diagnosed with benign conditions following resection for suspected pancreatic cancer. Size of squares proportion reflects the weight of the trial in the pooled analysis. The diamonds show the pooled prevalence of the types. Horizontal bars represent 95% CI. Red lines show the prediction interval

The type 1 AIP proportion within benign cases was 0.45 (95% CI: 0.11–0.85), with moderate diversity (heterogeneity: 67%, CI: 33-84%). The prediction interval was 0 to 0.99. The type 2 AIP proportion was 0.07 (95% CI: 0.03–0.16), with no heterogeneity (0%, CI: 0-75%). The prediction interval was 0.03 to 0.17. A statistical test indicated a significant difference between type 1 and type 2 AIP within benign scenarios (*p* = 0.007). (See Fig. 3).

The male/female ratio showed that AIP has a higher prevalence in males at 70%, compared to 30% in females. The mean age for AIP patients was 59 ± 7.5 years.

Based on pre-operative serological findings, serum IgG4 levels were elevated in 43% (21/49) of type 1 AIP patients. Further, 23 out of 47 (49%) AIP patients had elevated CA 19 − 9 concentrations (average 3720 ± 8646 U/mL), where higher levels were found in those with type 1 AIP (22/43 patients, 51%) compared to type 2 AIP (1/4 patients, 25%).

**Fig. 4:**
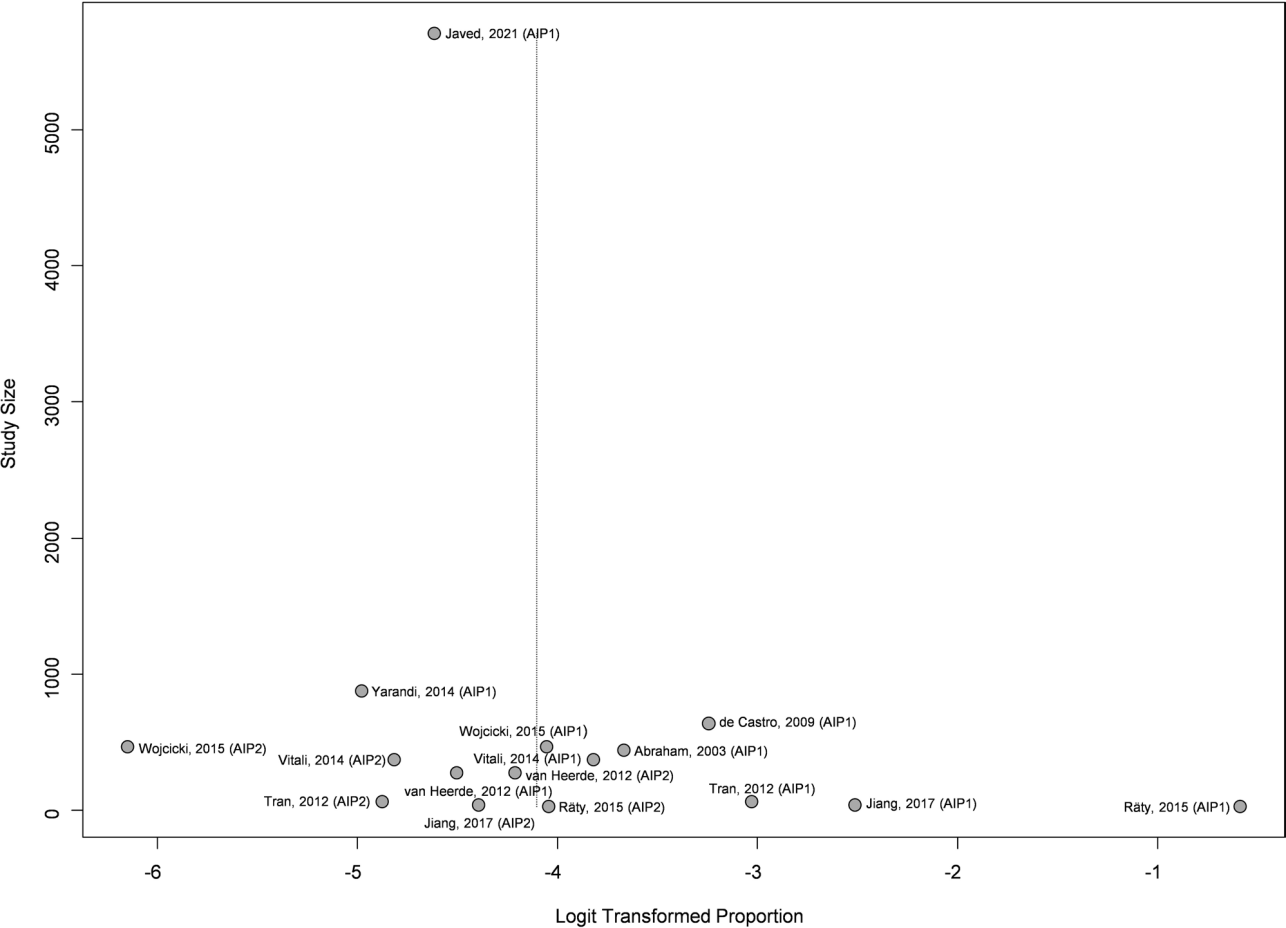
Funnel plot of the studies included for the meta-analysis of the prevalence of type 1 and type 2 autoimmune pancreatitis in patients undergoing resection due to suspected pancreatic cancer. The funnel plot shows the logit proportion (horizontal axis) against the study size (vertical axis)

**Fig. 5:**
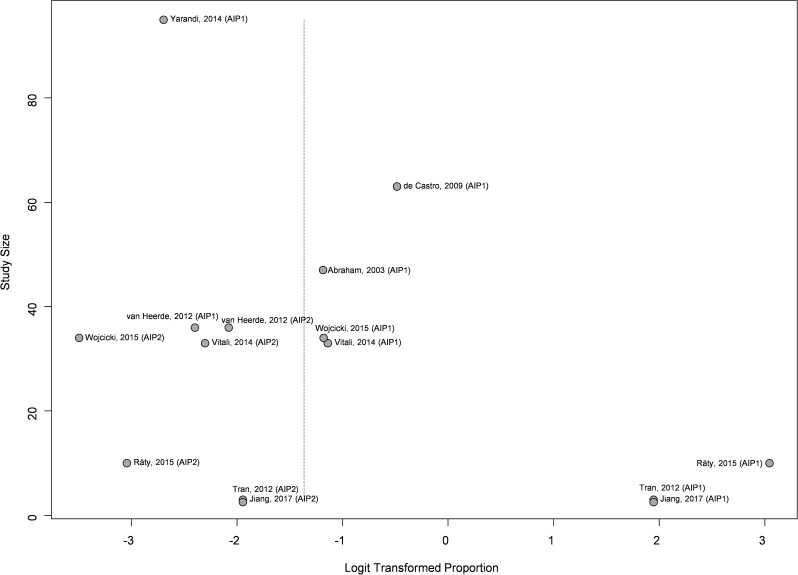
Funnel plot of the studies included for the meta-analysis of the prevalence of type 1 and type 2 autoimmune pancreatitis in patients diagnosed with benign conditions following resection for suspected pancreatic cancer. The funnel plot shows the logit proportion (horizontal axis) against the study size (vertical axis)

Publication bias was observed (*p* = 0,004) for assessment of the prevalence of type 1 and type 2 autoimmune pancreatitis in patients undergoing resection due to suspected pancreatic cancer; one study was detected as outlier (Räty, 2015 (AIP1). No publication bias was observed (*p* = 0,704) for the assessment of the prevalence of type 1 and type 2 autoimmune pancreatitis among patients diagnosed with benign conditions following resection for suspected pancreatic cancer (See Figs. 4 and 5).

## Discussion

In the present study, we found that 324 out of 3208 (10.1%) patients where the histological examination of the resected specimen revealed a benign pancreatic lesion were scheduled to undergo a pancreatectomy. This incidence falls within the interval of 5–11% reported in the literature [21]. AIP accounted for 1.6% of all suspected cases involving a surgical procedure and was responsible for the most significant subset (25.9%) of benign disease. This incidence is in agreement with results reported by J. Wojcicki et al. (26.5%) [20] and S. Abraham et al. (27.5%) [13].

The Honolulu Consensus Document divides AIP into two subclasses [16], which differ in their histological patterns and clinical presentations. Our study showed a dominant prevalence of type 1 (94%) compared with type 2 (6%). This result is also in agreement with the literature [22]. However, based on the available data, the mean age of the AIP patients was 58.9 years, which was relatively lower than that reported by J. Hardacre et al. (62 years) [23] and T. Kamisawa et al. (66.3 years) [22]. Our male-to-female ratio (3.1) was consistent with recent epidemiological data [24].

Based on the included studies, patients with a final diagnosis of pancreatic malignancy were significantly older than those with benign disease (at least five years older) [6, 18]. Interestingly, S. De Castro et al. reported a significant difference in the male/female ratio between patients with pancreatitis and patients with pancreatic adenocarcinoma [6].

S. Yarandi et al. presented an analysis of findings in benign patients compared to those with pancreatic cancer, demonstrating that an odds ratio of alcohol abuse as a risk factor for pancreatitis was significantly higher in patients with benign diseases [21]. Pain as the main symptom in patients with benign diseases occurred significantly more than those with PDAC [6, 21].

There are several imaging techniques, characteristic pancreatic morphological features, a serum biomarker to distinguish AIP from PDAC [25–28]. However, more than 30% of AIP patients will require pancreatic core biopsy to make the diagnosis [25].

Most of the cited studies presented the radiologic work-up for patients submitted to surgery. Despite the use of a variety of radiologic techniques, such as CT, ERCP, MRI and EUS, radiology was ultimately sufficiently indicative or non-diagnostically compelling for surgeons to opt for an operation in almost all cases.

Wojcicki et al. conducted a retrospective analysis, comparing pre-operative diagnoses, revised radiological diagnoses, and final histology results in 21 cases. They found that the most common missed diagnoses were benign conditions affecting the distal common bile duct, the pancreaticoduodenal groove, and AIP. The reviewers were able to retrospectively determine the correct diagnosis in almost half of the cases (10 out of 21) based solely on the radiological images. It is important to note that initial radiology reports identified a mass in 20 out of 34 cases (59%), while only 3 cases out of 21 (14%) showed a mass after reviewing the images [20]. Van Heerde et al. [18] mentioned that all seven patients with AIP had a sufficient suspicion index to justify the operation including: significantly elevated Ca19-9 levels (reaching as high as 23,284 kU/l), suggestive imaging findings (such as a mass on EUS, double duct sign on CT/MRI or ERCP, as well as false positive cytology results from (EUS-FNA). However, chronic pancreatitis was suspected in nearly a third of patients in a study of Javed et al. [14] but could not be definitively diagnosed. In approximately 16% of patients, radiological findings suggested AIP. However, among these patients, 88.9% had a dilated main pancreatic duct, 33.4% had elevated CA 19 − 9 levels, and 55.6% did not have elevated IgG4 levels.

The use of serological biomarkers can be essential in differentiating AP from PDAC. The most commonly used biomarkers in pancreatic pathology are CA 19 − 9 and IgG4. Since type 1 AIP is characterised histologically by an infiltrate of IgG4 positive plasma cells, serum IgG4 level is often elevated. However, the literature did report cases with normal IgG4 levels [29]. In our study, based on available data, pre-operative serological findings showed that only 21 out of 49 patients (43%) with type 1 AIP had elevated serum levels of IgG4.

Measuring IgG4 serum level is recommended when IgG4 disease is suspected; however, on its own, it lacks sensitivity and specificity [16]. The sensitivity in our study (43%) is surprisingly low, which was not consistent with the results of a recent meta-analysis which showed that the sensitivity of IgG4 is 72% [30]. The reference range in our study for IgG4 was 3.9–86.4 mg/dL, and the average concentration of elevated levels was 324 ± 99 mg/dL. All patients with a high concentration of IgG4 underwent surgical treatment, and the final diagnosis was AIP. In a recent study included in our review [18], only 25% of AIP patients operated on for suspicion of PDAC had an elevated serum IgG4 level. In addition, serum IgG4 elevation may occur in 10% of patients with PDAC [31]; it, therefore, cannot be used as a tool for distinguishing AIP from PDAC.

CA 19 − 9 is widely known as a biomarker for PDAC with a sensitivity of 79–95% and a specificity of 82–91% [32]. Studies reported that CA 19 − 9 can also be elevated in benign conditions in the hepatobiliary system, lungs and kidneys [32]. Many case series have also shown high serum levels of CA 19 − 9 in patients suffering from AIP [33–35]. We found that CA 19 − 9 levels were elevated in 51.1% of patients with type 1 AIP and in 25% of those with type 2 AIP. This range is higher than those (27–36%) in the literature [24, 26, 27]. However, as many as 51.6% (16/31) of AIP patients operated on for suspicion of PDAC had an elevated serum CA 19 − 9 level in a recent study, which is in line with our results [14].

The amplitude of the elevation was very high, almost 100 times over the upper limit of the normal range (37 U/mL) in our study. In fact, one explanation for this high amplitude could be the extreme values of CA 19 − 9 in two patients, whose symptoms and radiology were strongly suggestive of neoplasm and whose CA 19 − 9 levels were 23,284 U/mL and 1689 U/mL. The final diagnosis for those patients were AIP type 1 and AIP type 2, respectively [18]. However, the literature reported very high (> 12,000 U/mL) elevation of serum CA 19 − 9 in patients who had undergone pancreatic surgery for a benign disease, where surgery was unavoidable even after applying the ICDC criteria [33]. Unfortunately, no data were available in the ten included studies on the levels of CA 19 − 9 in patients with PDAC to compare to those with AIP.

There is therefore a pressing need to identify reliable biomarkers to differentiate between PDAC and AIP. Thus, further studies are crucial in the future to help find more accurate diagnostic tools to detect non-neoplastic diseases before performing unnecessary surgery. Until then, combined serum IgG4 and CA19-9 measurement [24] and EUS-guided fine needle biopsy are the main diagnostic tools to differentiate AIP from PDAC. Unfortunately, only two studies mentioned the results of a pre-operative EUS-FNA. FNA samples were obtained for five patients in a study by Wojcicki et al. with a result of four benign cells and one atypical one [20]. The other study reported findings from 35 patients as non-diagnostic (18 patients), with PDAC (11 patients) and with chronic pancreatitis (four patients) as well as three patients with cellular atypia [14].

Nowadays, neoadjuvant therapy has been widely recommended for managing patients with borderline resectable pancreatic cancer and resectable tumors with high risk factors. Therefore, preoperative tissue sampling of resectable pancreatic masses is more frequently recommended. Indeed, preoperative EUS-FNA and neoadjuvant therapy in resectable pancreatic cancer is associated with significantly greater OS when compared to the upfront surgery group, with no significant difference in the rates of tumor recurrence or peritoneal seeding [36, 37] The strategy performing EUS-FNA is all resectable pancreatic cancer, may avoid misdiagnosing AIP in the future.

A key strength of our meta-analysis is that most studies included a representative initial population and complete histological reporting of the final diagnosis after surgery. However, a weakness is that this meta-analysis was based on 10 studies, all of which were observational studies, precluding a low certainty of evidence. One study was detected as outlier (Räty, 2015) in the publication bias analysis, reporting high number (10%) of AIP patients in their cohort. Furthermore, four studies were of low quality (Newcastle-Ottawa scale < 4). These studies exhibited significant disparities in group comparability, particularly between male/female patients with AIP and in age between AIP and PDAC patients. Nevertheless, these data did not influence the outcome of our meta-analysis.

## Conclusions

In conclusion, our findings underscore the intricacies in diagnosing benign pancreatic lesions and differentiating these conditions from pancreatic malignancies. Despite modern diagnostic methods, unnecessary surgery cannot be avoided in some benign patients, among whom a diagnosis of AIP was responsible for almost one third. Serum CA 19 − 9 or IgG4 is unable to differentiate AIP from PDAC. Further research and the development of more precise diagnostic tools are imperative to prevent unnecessary surgeries and improve patient outcomes in the context of pancreatic diseases.

## Data Availability

All data generated or analysed during this study are included in this published article.

## Abbreviations

AIP: Autoimmune Pancreatitis
CA 19 − 9: Carbohydrate Antigen 19 − 9
CT: Computed Tomography
ERCP: Endoscopic Retrograde Cholangiopancreatography
EUS: Endoscopic Ultrasound
FNA: Fine Needle Aspiration
IDCP: Intraductal Papillary Mucinous Neoplasm
LPSP: Lymphoplasmacytic Sclerosing Pancreatitis
MRI: Magnetic Resonance Imaging
PDAC: Pancreatic Ductal Adenocarcinoma
